# Frequency of Heterozygous Parkin (*PRKN*) Variants and Penetrance of Parkinson's Disease Risk Markers in the Population-Based CHRIS Cohort

**DOI:** 10.3389/fneur.2021.706145

**Published:** 2021-08-09

**Authors:** Maria Paulina Castelo Rueda, Athina Raftopoulou, Martin Gögele, Max Borsche, David Emmert, Christian Fuchsberger, Essi M. Hantikainen, Vladimir Vukovic, Christine Klein, Peter P. Pramstaller, Irene Pichler, Andrew A. Hicks

**Affiliations:** ^1^Institute for Biomedicine, Eurac Research, Affiliated Institute of the University of Lübeck, Bolzano, Italy; ^2^Department of Economics, University of Patras, Patras, Greece; ^3^Institute of Neurogenetics, University of Lübeck, Lübeck, Germany; ^4^Centre for Disease Control and Prevention, Institute of Public Health of Vojvodina, Novi Sad, Serbia; ^5^Department of Neurology, University Medical Center Schleswig-Holstein, Lübeck, Germany

**Keywords:** Parkin, heterozygote mutation, Parkinson's disease, risk markers, penetrance, population

## Abstract

Mutations in the *Parkin* (*PRKN*) gene are the most frequent cause of autosomal recessive early-onset Parkinson's disease (PD). Heterozygous *PRKN* mutation carriers might also be at increased risk for developing clinical symptoms of PD. Given the high frequency of heterozygous mutations in the general population, it is essential to have better estimates of the penetrance of these variants, and to investigate, which clinical and biochemical markers are present in carriers and thus potentially useful for identifying those individuals at greater risk of developing clinical symptoms later in life. In the present study, we ascertained the frequency of heterozygous *PRKN* mutation carriers in a large population sample of the Cooperative Health Research in South Tyrol (CHRIS) study, and screened for reported PD risk markers. 164 confirmed heterozygous *PRKN* mutation carriers were compared with 2,582 controls. A higher number of heterozygous mutation carriers reported a detectable increase in an akinesia-related phenotype, and a higher percentage of carriers had manifested diabetes. We also observed lower resting heart rate in the *PRKN* mutation carriers. Extending our risk analyses to a larger number of potential carriers and non-carriers using genotype imputation (*n* = 299 carriers and *n* = 7,127 non-carriers), from previously published biomarkers we also observed a higher neutrophil-to-lymphocyte ratio (NLR) and lower serum albumin and sodium levels in the heterozygous *PRKN* variant carriers. These results identify a set of biomarkers that might be useful either individually or as an ensemble to identify variant carriers at greater risk of health issues due to carrier status.

## Introduction

Parkinson's disease (PD) is one of the most common neurodegenerative diseases in aging, second only to Alzheimer's disease (1). Most PD cases are classified idiopathic (~90%), and about 10% of cases represent rare forms with Mendelian inheritance patterns, ascribable to rare mutations in specific genes, with more than 20 identified to date (2). However, many of these genes lack replication and their relevance is therefore still debated. In addition, genome-wide association studies have uncovered ~90 independent risk signals in 78 genomic loci at genome-wide significance, that contribute to genetic risk for sporadic PD (3). Mutations in *PRKN* (the gene that encodes Parkin) are the most common known cause of autosomal recessive early-onset PD, accounting for up to 42.2% of cases with an age of onset ≤20 years (4), and Parkin dysfunction represents a risk factor for sporadic PD (5). *PRKN* variants have been identified in numerous families with different genetic background (6) and include more than 130 mutations consisting of copy number variations (CNVs) (deletions or multiplications of exons), small deletions/insertions, as well as single nucleotide polymorphisms (SNPs) (missense, non-sense or splice side variations) (7, 8). Importantly, a large number of patients carry exon rearrangements that result in protein truncation (8, 9). The Parkin protein functions as an E3 ubiquitin ligase, which is implicated in the clearance of dysfunctional mitochondria by autophagy (mitophagy) and in several other cellular processes like mitochondrial biogenesis, free radical metabolism, and inflammation.

Homozygous or compound-heterozygous mutations in the *PRKN* gene result in highly penetrant symptom expression, while heterozygous mutations may predispose to disease symptoms with highly reduced penetrance (10, 11), with a similar pattern established for mutations in *GBA* (12, 13). Heterozygous *PRKN* mutations are found more frequently in PD patients than in controls (14), where they are detected with a frequency up to 3.1% (15). The presence of heterozygous *PRKN* mutations is one of the reported potential genetic risk factors for PD (10, 14, 15). Given the frequency (up to 3%) of heterozygous mutations in the population, it is essential to have better estimates of the penetrance of these variants and to investigate, which risk markers are manifesting in carriers and thus potentially useful for identifying those individuals at greater risk of clinical symptoms later in life. In the present study, we ascertained the frequency of heterozygous *PRKN* mutation carriers in a large population sample of the Cooperative Health Research in South Tyrol (CHRIS) study conducted in Northern Italy (16), in the same geographic region, where the largest pedigree to date with *PRKN* mutations was identified (17). We also screened for validated PD risk markers of the *Movement Disorder Society (MDS)* (18) and additional potential PD risk markers reported in the literature (19–22).

## Methods

### Study Participants

The CHRIS study (16) is a longitudinal population-based study to investigate the genetic and molecular basis of age-related common chronic conditions and their interaction with genes, lifestyle and environment in the general population. All adults of one valley in South Tyrol (Northern Italy) were invited, with 13,393 participants enrolled. Of these, 10,500 were genotyped with either the OmniExpressExome chip (Illumina), that contains 958,497 markers including over 273,000 functional exome markers, or with the Omni2.5Exome chip (Illumina) with 2,618,000 markers that include over 240,000 exonic markers. Genotypes were imputed (23) to 39.2 million variants using the Haplotype Reference Consortium panel (http://www.haplotype-reference-consortium.org). Exome sequencing was performed using the xGen® Exome Research Panel v1.0 in 3,603 individuals. Family participation was encouraged for complete pedigree reconstruction. The study was approved by the Ethics Committee of the Healthcare System of the Autonomous Province of Bozen/Bolzano (Italy).

### Phenotypes

In this study, PD risk markers of the *Movement Disorder Society (MDS) research criteria for prodromal PD* were assessed (18). All risk markers, except for occupational solvent exposure and *substantia nigra* hyperechogenicity, were available for analysis. Significant pesticide exposure was assessed by a computer-assisted interviewer-administered question “Do you use pesticides? (insecticides, herbicides, fungicides).” The non-use of caffeine was defined as the response “rarely or never” (in the past 12 months) to the item “Coffee (mocha/espresso, filter coffee, instant)” of the GA2LEN food frequency questionnaire (24). Physical activity was assessed by using the International physical activity questionnaire (25), and life-course smoking was measured by the European Community Respiratory Health Survey (26). Diabetes was defined as either self-reported doctor-diagnosed and/or use of glucose-lowering drugs or fasting serum glucose ≥126 mg/dL or glycated hemoglobin (HbA1c) ≥6.5%. Additional potential PD risk markers were available for analysis: C-reactive protein (CRP) (21), Platelet count (PC), Mean platelet volume (MPV) (20), Platelet to lymphocyte ratio (PLR), Neutrophil to lymphocyte ratio (NLR) (22), heart rate, systolic blood pressure (BP), white blood cell (WBC), neutrophil counts, serum albumin, sodium (19). Blood samples were drawn after overnight fasting and blood parameters measured by standard methods (16). For evaluating PD symptoms, a validated screening questionnaire composed of nine symptom questions and two additional questions about the patient's diagnosis of parkinsonism and/or treatment was used (27).

### Mutation Analysis

To search for predicted CNVs over the *PRKN* gene, we used the SNP & Variation Suite software (SVS version 8, Golden Helix) to examine the signal intensity information (LogR) from available chip genotyping data. To confirm predicted deletions or duplications of specific exons in the gene, DNA was further analyzed by Multiplex ligation-dependent probe amplification (MLPA) or digital PCR assays (ddPCR). In addition to the detection of deletions/duplications over exons of the *PRKN* gene, we used an in-house instance of the BRAVO (Browse All Variants Online) browser, which is a web-based browser for genetic variation determined in the whole exome sequencing dataset of 3,603 CHRIS participants. All variants were annotated with the Ensembl Variant Effect Predictor (VEP) and the Combined Annotation Dependent Depletion (CADD) algorithms (28, 29). We decided a CADD score cutoff of >20, which was more likely to capture predicted pathogenic variants by comparison with variants described in the MDSGene database (https://www.mdsgene.org/). We used these resources and databases to identify carriers of pathogenic or likely pathogenic point mutations and small deletions/insertions in our exome sequence and imputed chip genotyping datasets. Additionally, published or suspected pathogenic variants in 18 PD and dystonia-related genes other than *PRKN* were identified, and individuals carrying such variants were excluded from the control set used for the exome sequence dataset (resulting in *n* = 2,582 controls) and the imputed dataset (resulting in 7,127 controls).

### Statistical Analysis

Mann-Whitney and Fisher's exact tests were used to compare non-normally distributed variables per heterozygous *PRKN* mutation carrier status. Analyses were conducted in Stata version 15.1 (StataCorp, College Station, TX, USA).

## Results

In the subset of the CHRIS study, for which exome sequence data are available (*n* = 3,603 participants), 164 individuals carried one of 17 known or newly detected heterozygous mutations in *PRKN* resulting in a carrier frequency of 4.55%. Large exon rearrangements affecting exons 2, 3, 4, and 7 of the *PRKN* gene were identified and confirmed in 31 individuals (0.86%). Two small frameshift deletions (delA in exon 9, p.Val324AlafsTer111 - rs1562519380); del CT in exon 2, p.Gln34ArgfsTer5 - rs55777503) were detected in 35 individuals, and 98 individuals carried one out of 11 single nucleotide variations (eight known and three novel) resulting in a missense mutation. The most frequent mutation is the p.Arg275Trp missense mutation (rs34424986) with 1.64% (Table 1). Not surprisingly, a heterozygous deletion of exon 7 (*n* = 24 individuals, 0.67%) and the 1-bp deletion in exon 9 (*n* = 18 individuals, 0.50%), which are the two mutations identified in the previously described pedigree with *PRKN* mutations (17), are among the most frequent mutations identified. Heterozygous *PRKN* mutation carriers were clustered in eight multigenerational families and four small multiplex families. Most pedigrees with either the exon 7 deletion or the 1-bp deletion in exon 9 could be connected to the large pedigree with *PRKN* mutations, further pointing to a founder effect for these two mutations (6).

**Table 1:** Heterozygous mutations in the *PRKN* gene identified in the exome sequenced dataset of the CHRIS cohort (*n* = 3,603).

***PRKN* mutation(DNA change/rsID)**	**Consequence**	**CADD score**	**Number of carriers (*n* = 164)**	**Carrier frequency (%)**	**Allele frequency (%)**	**gnomAD allele frequency (%)**
Exon 7 deletion	CNV, frameshift	/	24	0.67	0.33	/
Exon 2 deletion	CNV	/	4	0.11	0.06	/
Exon 2,3,4 duplication	CNV	/	2	0.06	0.03	/
Exon 4 duplication	CNV	/	1	0.03	0.014	/
GA/G (rs1562519380)	Frameshift, p.Val324AlafsTer111	33	18	0.50	0.25	/
CCT/C (rs55777503)	Frameshift, p.Gln34ArgfsTer5	32	17	0.47	0.23	0.02
G/A (rs34424986)	Missense, p.Arg275Trp	30	59	1.64	0.82	0.19
G/A (rs150562946)	Missense, p.Arg256Cys	32	11	0.30	0.14	0.04
C/T (rs146173584)	Missense, p.Asp243Asn	24	10	0.28	0.14	0.026
C/T (rs748892763)	Missense, p.Arg104Gln	23	6	0.17	0.08	0.0012
G/A (rs55830907)	Missense, p.Arg402Cys	24	3	0.08	0.04	0.18
G/A (rs137853054)	Missense, p.Thr240Met	24	3	0.08	0.04	0.034
G/C	Missense, p.Cys441Trp	29	2	0.06	0.03	/
G/A (rs149953814)	Missense, p.Pro437Leu	28	1	0.03	0.014	0.15
T/G (rs182893847)	Missense, p.Met458Leu	24	1	0.03	0.014	0.028
T/C	Missense, p.Tyr267Cys	28	1	0.03	0.014	/
C/T (c.8-1G>A)	Splice acceptor	35	1	0.03	0.014	/

*CNV, copy number variant*.

*CADD (Combined Annotation Dependent Depletion): tool for scoring the deleteriousness of single nucleotide variants as well as insertion/deletion variants in the human genome; variants with a score of >20 were taken into consideration in our study (28, 29)*.

*gnomAD: Genome Aggregation Database (https://gnomad.broadinstitute.org/)*.

The analysis of the PD screening questionnaire revealed that a higher number of heterozygous carriers of a *PRKN* mutation reported that “*their feet seem to get stuck to the floor*” (symptom question 5) (*p* = 0.006, *alpha set at 0.0045 after Bonferroni correction*) as compared to non-carriers, indicating a detectable increase in an akinesia-related phenotype (Supplementary Table 1). The 164 heterozygous *PRKN* mutation carriers were further compared with 2,582 controls in terms of risk markers associated with PD. The descriptive analysis for all MDS risk markers (18), except for occupational solvent exposure and *substantia nigra* hyperechogenicity, is presented in Table 2, reporting percentages for the binary variables and means, medians, and inter-quartile range (IQR) for the continuous variables. Interestingly, a higher percentage of heterozygous *PRKN* mutation carriers manifest diabetes mellitus (*p* = 0.023), which is known to be associated with PD risk in multiple population-based prospective studies (18). In addition to the established PD risk markers, we included potential PD risk markers in our analyses, which we identified based on recent data in the literature. For these markers, we observed a trend toward a lower heart rate in the *PRKN* mutation carriers (59.01 vs. 60.6, *p* = 0.054) as compared to the controls. When extending our risk marker analyses to the imputed *PRKN* carrier dataset (*n* = 299 carriers and *n* = 7,127 non-carriers, Supplementary Table 2), we also found higher neutrophil-to-lymphocyte ratio (NLR) (4.91 vs. 4.71, *p* = 0.025) and lower serum albumin (4.45 vs. 4.49, *p* = 0.008) and sodium levels (140.22 vs. 140.44, *p* = 0.047) in the *PRKN* variant carriers (Supplementary Table 3).

**Table 2:** Descriptive statistics of PD risk markers in heterozygous *PRKN* mutation carriers in the exome sequenced dataset of the CHRIS study.

**PD risk markers**	**Controls (** ***n*** **=** **2,582)**							**Mutation carriers (** ***n*** **=** **164)**							***p-*value**
Sex, *n* (%)															
Male	1,148 (44.46)							68 (41.46)							0.454
Female	1,434 (55.54)							96 (58.54)							
Pesticide exposure, *n* (%)															
Yes	497 (27.25)							30 (25.86)							0.745
No	1,327 (72.75)							86 (74.14)							
Non-use of caffeine, *n* (%)															
Yes	40 (15.21)							2 (12.50)							0.769
No	223 (84.79)							14 (87.50)							
Non-smoker, *n* (%)															
Yes	1,973 (80.79)							126 (80.77)							0.994
No	469 (19.21)							30 (19.23)							
Diabetes, *n* (%)															
Yes	119 (4.85)							14 (8.97)							**0.023**
No	2,335 (95.15)							142 (91.03)							
	***n***	**Mean**	**SD**	**Median**	**IQR (25–75)**	**Min**	**Max**	***n***	**Mean**	**SD**	**Median**	**IQR (25–75)**	**Min**	**Max**	***p-*** **value**
Age (years)	2,582	46.03	16.66	46.37	25.94	18.03	93.51	164	44.58	16.85	43.45	29.34	18.02	87.71	0.2948
Uric acid (mg/dL)	2,582	5.14	1.35	5	1.9	1.4	11	163	5.23	1.30	5.2	1.9	2.4	8.4	0.2209
Physical activity (MET-*minutes*/*week*)	2,455	1,376	955	1,260	1,505	0	3,780	151	1,444	948.89	1,260	1,610	0	3,360	0.3336
CRP (mg/dL)	2,416	0.24	0.38	0.13	0.21	0	7.15	152	0.28	0.48	0.11	0.245	0	3.46	0.7314
PC (x1,000/μL)	2,580	259.52	57.52	254	74	73	516	163	267.92	73.57	257	78	149	732	0.5662
MPV (fL)	2,580	8.7	1.41	8.5	2	4.3	13.7	163	8.62	1.37	8.4	2.1	5.5	12.4	0.4906
PLR (ratio)	2,580	8.09	2.94	7.60	3.30	1.79	39.83	163	8.23	3.05	7.47	3.11	3.35	21.08	0.8147
NLR (ratio)	2,580	4.87	1.35	4.69	1.63	1.26	17.90	163	5.03	1.55	4.88	1.56	2.12	15.77	0.2677
Heart rate (bpm)	2,562	60.60	9.73	60	12	34	120	163	59.01	8.87	59	13	41	86	**0.0538**
Systolic BP (mmHg)	2,578	119.58	16.82	117	21	84	201	164	117.51	15.12	117	19	90	167	0.2730
WBC (x1,000/μL)	2,580	6.17	1.58	5.9	1.9	2.6	19.3	163	6.10	1.51	6	1.9	2.9	10.9	0.6535
Neutrophil counts (x1,000/μL)	2,580	3.42	1.24	3.2	1.4	0.5	15.4	163	3.36	1.15	3.2	1.5	1.6	8.5	0.5798
Serum albumin (g/dL)	2,582	4.59	0.30	4.6	0.40	3.3	5.9	163	4.57	0.33	4.6	0.5	3.5	5.6	0.1652
Sodium (mmol/L)	2,420	140.50	2.30	140	3	132	155	151	140.57	2.39	141	3	134	148	0.6593

*PD risk markers from Heinzel et al. (18) are underlined*.

*Physical activity was measured as metabolic equivalent of task (MET)-minutes per week*.

*Additional potential PD risk markers were chosen based on Qiu et al. (21): CRP, C-reactive protein; Kocer et al. (20): PC, Platelet count; MPV, Mean platelet volume; Sanjari Moghaddam et al. (22): PLR, Platelet to lymphocyte ratio; NLR, Neutrophil to lymphocyte ratio; Iwaki et al. (19): heart rate, systolic blood pressure (BP), white blood cell (WBC), neutrophil counts, serum albumin, sodium*.

*Data reported here are not corrected for multiple comparison*.

*After Bonferroni correction, alpha is set at 0.0026*.

## Discussion

Previous studies have estimated the frequency of rare, heterozygous *PRKN* mutations in healthy control individuals up to 3.2%, with a previous South Tyrolean sample showing a frequency of 3.9%, and two studies that identified no mutation in healthy control individuals (30). We have screened 3,603 individuals of the population-based CHRIS study, free of overt clinical symptoms related to movement disorders, by exome sequencing and CNV analysis and have identified heterozygous mutations in 4.55% of the study participants (*n* = 164 individuals). Given the low residential mobility across generations in this restricted area of the Alps, rare variants might be overrepresented (31) as it can be seen also from the comparison with gnomAD allele frequencies (Table 1), and a founder effect can be assumed for some of the *PRKN* mutations identified in this study. This might explain the higher frequency compared to previous studies. However, the CNV carrier frequency of 0.86% detected in this study is in keeping with previously published reports (10, 32).

None of the mutation carriers identified fulfilled the clinical criteria for a PD diagnosis. However, a higher percentage of mutation carriers reported that their “feet seem to get stuck to the floor.” This specific symptom item might indicate subtle motor signs in an early phase of disease conversion. In support of this, we recently observed in a subset of 27 heterozygous *PRKN* mutation carriers and 24 non-mutation carriers from our study, who were recalled based on their genetic profile, that sensor-based quantification of movements allows discrimination between unaffected heterozygous mutation carriers and mutation-free controls. Thereby, it is crucial to challenge the motor system with more difficult tasks to unmask these subtle motor alterations (33). These results are further supported by a recent study, which found an increased risk of developing PD in heterozygous *PRKN* mutation carriers, although ~70% of putative heterozygous cases were not assessed for a second mutation (14). Pre-clinical changes identified by neuroimaging studies and a reduced fluorodopa uptake in the striatum previously observed in asymptomatic heterozygous *PRKN* mutation carriers also support an increased risk of developing PD symptoms in heterozygous *PRKN* mutation carriers (34, 35).

Diabetes has recently been defined as a novel PD risk marker as part of *The MDS Research Criteria for Prodromal PD* with a positive and negative likelihood ratio of 1.5 and 0.97, respectively (18). Furthermore, a recent meta-analysis found an association of type-2 diabetes with PD and some evidence for an increased progression of motor symptoms and cognitive decline (36). First studies showed a potential effect of anti-diabetic drugs on the progression of PD (37). Notably, diabetes was associated with the heterozygous *PRKN* mutation carrier status in our study. In this context, it has recently been shown that metformin, an anti-diabetic drug, which induces mild mitochondrial stress, phosphorylates and thereby activates endogenous Parkin in the liver, suggesting that Parkin plays an important role in mitochondrial homeostasis in this physiological context (38).

Furthermore, a previous study has found a higher heart rate in PD subjects (19), while we observed a tendency for a lower heart rate in our *PRKN* carriers. A recent study performed in cardiomyocytes and sera derived from PD patients with biallelic *PRKN* mutations found no alterations in Troponin T levels, suggesting that Parkin deficiency may not be associated with cardiac damage (39). However, animal studies suggested that Parkin might protect from cardiac damage (40, 41).

Moreover, when extending our analysis to the larger dataset with imputed genotypes, we also identified three molecular markers, which are different between putative mutation carriers and non-carrying controls. The peripheral immune biomarker NLR, which is increased in this group of mutation carriers, was previously found to be higher in idiopathic PD patients as compared to controls (42). Although there is one contradicting study for this association (43), it has been recently confirmed by Iwaki et al. (19). Interestingly, for NLR, a negative correlation to striatal binding ratios of DAT SPECT images in bilateral caudate and putamen nuclei of drug-naïve early PD patients was observed, and a positive association of NLR and motor severity was detected in the tremor-dominant subgroup of PD patients (22). Serum albumin and sodium are decreased in the *PRKN* mutation carriers of our enlarged dataset, in contrast to a previously found association with PD (19).

Carrying heterozygous *PRKN* mutations is one of the reported potential genetic risk factors for PD, and therefore identifying those individuals at possibly greater risk of disease development requires identification of biological markers that can be monitored in the prodromal phase. While data is lacking about conversion of individuals carrying such genetic risk factors to full disease, it is crucial to identify such markers, as these will be vital for testing potential neuroprotective therapies before progressive neurodegeneration advances, as it has been shown in individuals at high risk for type I diabetes (44). While we have identified associations for several risk markers, they cannot be used to predict an individual risk to develop PD. The prodromal phase of PD is clinically characterized not only by the presence of non-motor signs but also subtle motor disturbances as part of the phenotypic spectrum. To adequately understand individual disease conversion, there is a need for more longitudinal studies with individuals at greater risk and larger sample sizes using more in-depth phenotyping such as analysis of gait (33, 45). Here, we showed that heterozygous *PRKN* mutation carriers in the general population may self-report subtle motor signs (which can then be followed up by deeper phenotyping), have increased occurrence of diabetes, decreased heart rate, alterations of serum albumin and sodium, and increased NLR ratios, potentially further pointing to inflammatory activation as an early sign in the development of *PRKN*-related PD (46).

## Data Availability Statement

The datasets presented in this report are not readily available because the data are part of a large population-based study, and the informed consent provided by the study participants does not allow the upload of individual-level genetic data to public repositories. Requests to access the datasets (including individual-level genetic data) should be directed to the Eurac data access committee (http://chris.eurac.edu).

## Ethics Statement

The study was approved by the Ethics Committee of the Healthcare System of the Autonomous Province of Bozen/Bolzano (Italy). The patients/participants provided their written informed consent to participate in this study.

## Author Contributions

MPCR performed ddPCR experiments. AR performed the statistical analyses. MG organized the clinical data collection. MB performed MLPA experiments. DE and CF performed the genotype imputation. EMH extracted the genotypes. VV elaborated the clinical data. CK and PP provided critical review of the manuscript. IP wrote the manuscript and organized the study. AH wrote the manuscript, designed, and organized the study. All authors contributed to the article and approved the submitted version.

## Conflict of Interest

The authors declare that the research was conducted in the absence of any commercial or financial relationships that could be construed as a potential conflict of interest.

## Publisher's Note

All claims expressed in this article are solely those of the authors and do not necessarily represent those of their affiliated organizations, or those of the publisher, the editors and the reviewers. Any product that may be evaluated in this article, or claim that may be made by its manufacturer, is not guaranteed or endorsed by the publisher.
