# Supplementary material for: Frequency of Heterozygous Parkin (PRKN) Variants and Penetrance of Parkinson's Disease Risk Markers in the Population-Based CHRIS Cohort
Source: Front Neurol. 2021 Aug 9;12:706145. doi: 10.3389/fneur.2021.706145 (PMC8382284; doi:10.3389/fneur.2021.706145)
Supplement: Supplementary file 1 [file Data_Sheet_1.docx]

Supplementary Material

**Table S1.** Analysis of PD screening questionnaire

|  | **CONTROLS n, (%)** | **MUTATION CARRIERS n, (%)** | ***p-value*** |
| --- | --- | --- | --- |
| **PDS1:** *Trouble arising from a chair* | 67 (2.60)  2,509 (97.40) | 4 (2.44)  160 (97.56) | 0.899 |
| **PDS2.** *Handwriting smaller than once* | 161 (6.35)  2,373 (93.65) | 12 (7.50)  148 (92.50) | 0.566 |
| **PDS3:** *Voice is softer than once* | 66 (2.57)  2,501 (97.43) | 4 (2.47)  158 (97.53) | 0.937 |
| **PDS4:** *Poor balance* | 131 (5.10)  2,439 (94.90) | 9 (5.49)  155 (94.51) | 0.826 |
| **PDS5:** *Feet seem to get stuck to the floor* | 30 (1.17)  2,542 (98.83) | 6 (3.68)  157 (96.32) | **0.006** |
| **PDS6:** *Face seems less expressive than once* | 33 (1.28)  2,542 (98.72) | 1 (0.61)  163 (99.39) | 0.451 |
| **PDS7:** *Arms or legs shake* | 85 (3.30)  2,489 (96.70) | 4 (2.44)  160 (97.56) | 0.546 |
| **PDS8:** *Trouble fastening buttons* | 128 (4.98)  2,444 (95.02) | 4 (2.45)  159 (97.55) | 0.145 |
| **PDS9:** *Shuffle or take small steps when you walk* | 118 (4.62)  2,437 (95.38) | 9 (5.59)  152 (94.41) | 0.571 |
| **PDS10:** *Parkinson’s disease diagnosis* | 67(2.60)  2,509 (97.40) | 4 (2.44)  160 (97.56) | 0.899 |
| **PDS11:** *Parkinson’s disease medication* | 161 (6.35)  2,373 (93.65) | 12 (7.50)  148 (92.50) | 0.566 |

PDS1-PDS11: PD screening questionnaire, symptom items 1-9 + two additional questions (Pramstaller et al. 1999).

After Bonferroni correction, alpha is set at 0.0045.

**Table S2.** Heterozygous mutations in the *PRKN* gene identified in the imputed dataset of the CHRIS cohort (n=10,500)

| ***PRKN* mutation**  **(DNA change / rsID)** | **Consequence** | **CADD score** | **Number of carriers (n=299)** | **Carrier frequency (%)** | **Allele frequency (%)** | **gnomAD allele frequency (%)** |
| --- | --- | --- | --- | --- | --- | --- |
| Exon 7 deletion | CNV, frameshift | / | 73 | 0.70 | 0.35 | / |
| Exon 2 deletion | CNV | / | 6 | 0.06 | 0.03 | / |
| Exon 2,3,4 duplication | CNV | / | 33 | 0.31 | 0.16 | / |
| Exon 2,3,4 deletion | CNV | / | 1 | 0.01 | 0.005 | / |
| Exon 4 duplication | CNV | / | 2 | 0.02 | 0.01 | / |
| Exon 3,4,5 deletion | CNV | / | 2 | 0.02 | 0.01 | / |
| G / A (rs34424986) | missense, p.Arg275Trp | 29 | 118 | 1.12 | 0.56 | 0.19 |
| G / A (rs150562946) | missense, p.Arg256Cys | 34 | 12 | 0.11 | 0.06 | 0.04 |
| G / A (rs55830907) | missense, p.Arg402Cys | 24 | 14 | 0.13 | 0.07 | 0.18 |
| G / A (rs137853054) | missense, p.Thr240Met | 24 | 3 | 0.03 | 0.01 | 0.034 |
| G / A (rs149953814) | missense, p.Pro437Leu | 29 | 35 | 0.33 | 0.17 | 0.15 |

# CADD (Combined Annotation Dependent Depletion): tool for scoring the deleteriousness of single nucleotide variants as well as insertion/deletion variants in the human genome; variants with a score of >20 were taken into consideration in our study (Kircher et al., 2014; McLaren et al., 2016)

gnomAD: Genome Aggregation Database (https://gnomad.broadinstitute.org/)

**Table S3.** Descriptive statistics of PD risk markers in heterozygous *PRKN* mutation carriers in the extended dataset with imputed genotypes of the CHRIS study

| PD risk markers | CONTROLS (n=7,127) | MUTATION CARRIERS (n=299) | *p-*value |
| --- | --- | --- | --- |
| Sex, n (%) |  |  |  |
| Male | 3,229 (45.31) | 129 (43.14) | 0.499 |
| Female | 3,898 (54.69) | 170 (56.86) |  |
| Pesticide exposure, n (%) |  |  |  |
| Yes | 1,109 (22.67) | 46 (22.77) | 0.973 |
| No | 3,783 (77.33) | 156 (77.23) |  |
| Non-use of caffeine, n (%) |  |  |  |
| Yes | 521 (13.30) | 24 (13.26) | 0.986 |
| No | 3,395 (86.70) | 157 (86.74) |  |
| Non-smoker, n (%) |  |  |  |
| Yes | 5,595 (82.06) | 220 (83.33) | 0.597 |
| No | 1,223 (17.94) | 44 (16.67) |  |
| Diabetes, n (%) |  |  |  |
| Yes | 297 (4.34) | 17 (6.37) | 0.113 |
| No | 6,554 (95.66) | 250 (93.63) |  |

|  | n | Mean | SD | Median | IQR (25-75) | Min | Max | n | Mean | SD | Median | IQR (25-75) | Min | Max | *p-*value |
| --- | --- | --- | --- | --- | --- | --- | --- | --- | --- | --- | --- | --- | --- | --- | --- |
| Age (years) | 7,127 | 45.86 | 16.27 | 46.44 | 25.06 | 18.00 | 93.51 | 299 | 44.64 | 15.71 | 44.08 | 24.53 | 18.23 | 82.76 | 0.249 |
| Uric acid (mg/dL) | 7,123 | 5.21 | 1.34 | 5.1 | 1.9 | 1.4 | 11.1 | 299 | 5.25 | 1.31 | 5.2 | 2 | 2.4 | 8.7 | 0.3936 |
| Physical activity (MET-minutes/week) | 6,612 | 3,909 | 3,473 | 2,880 | 4,473 | 0 | 19,278 | 281 | 3,938 | 3,580 | 2,970 | 4,716 | 0 | 17,838 | 0.9099 |
| CRP (mg/dL) | 6,851 | 0.27 | 0.48 | 0.14 | 0.23 | 0 | 17.24 | 262 | 0.32 | 0.52 | 0.14 | 0.28 | 0.01 | 3.66 | 0.0796 |
| PC (x1000/µL) | 7,120 | 253.54 | 56.80 | 248 | 70 | 46 | 856 | 281 | 261.81 | 64.75 | 257 | 68 | 139 | 732 | 0.0622 |
| MPV (fL) | 7,115 | 9.69 | 1.51 | 10 | 2.3 | 4.3 | 14.8 | 299 | 9.81 | 1.48 | 10.1 | 2.3 | 6 | 13 | 0.1707 |
| PLR (ratio) | 7,120 | 8.13 | 3.23 | 7.53 | 3.36 | 1.26 | 104.39 | 281 | 8.15 | 2.77 | 7.63 | 3.24 | 3.77 | 19.50 | 0.5625 |
| NLR (ratio) | 7,120 | 4.71 | 1.30 | 4.55 | 1.57 | 0.736 | 17.90 | 281 | 4.91 | 1.45 | 4.74 | 1.68 | 2.12 | 15.77 | **0.0245** |
| Heart rate (bpm) | 7,068 | 60.11 | 9.43 | 59.12 | 12 | 33 | 120 | 299 | 58.73 | 8.72 | 59 | 11 | 38 | 90 | **0.0218** |
| Systolic BP (mmHg) | 6,949 | 122.08 | 16.36 | 120 | 20 | 83 | 207 | 298 | 121.65 | 16.35 | 119 | 21 | 85 | 185 | 0.5821 |
| WBC (x1000/µL) | 7,120 | 6.12 | 1.67 | 5.9 | 1.9 | 1.5 | 47.8 | 299 | 6.07 | 1.43 | 5.9 | 1.8 | 2.9 | 10.8 | 0.9202 |
| Neutrophil counts (x1000/µL) | 7,120 | 3.42 | 1.26 | 3.2 | 1.4 | 0.5 | 17.8 | 299 | 3.34 | 1.07 | 3.2 | 1.4 | 1.3 | 8.5 | 0.7111 |
| Serum albumin (g/dL) | 7,125 | 4.49 | 0.29 | 4.5 | 0.39 | 3.2 | 5.9 | 299 | 4.45 | 0.30 | 4.4 | 0.29 | 3.5 | 5.4 | **0.0079** |
| Sodium (mmol/L) | 6,836 | 140.44 | 2.164 | 140 | 3 | 125 | 155 | 287 | 140.22 | 2.04 | 140 | 2 | 134 | 148 | **0.0468** |

PD risk markers from Heinzel et al. (Heinzel et al., 2019) are underlined.

Physical activity was measured as metabolic equivalent of task (MET)-minutes per week.

Additional potential PD risk markers were chosen based on Qiu et al. (Qiu et al., 2019): C-reactive protein (CRP); Kocer et al. (Kocer et al., 2013): Platelet count (PC), Mean platelet volume (MPV); Sanjari Moghaddam et al. (Sanjari Moghaddam et al., 2018): Platelet to lymphocyte ratio (PLR), Neutrophil to lymphocyte ratio (NLR); Iwaki et al. (Iwaki et al., 2020): heart rate, systolic blood pressure (BP), white blood cell (WBC), neutrophil counts, serum albumin, sodium.

Data reported here are not corrected for multiple comparison.

After Bonferroni correction, alpha is set at 0.0026.

# References

Heinzel, S., Berg, D., Gasser, T., Chen, H., Yao, C., Postuma, R.B., and Disease, M.D.S.T.F.o.t.D.o.P.s. (2019). Update of the MDS research criteria for prodromal Parkinson's disease. Mov Disord *34*, 1464-1470.

Iwaki, H., Leonard, H., Bandrés-Ciga, S., Blauwendraat, C., Scholz, S.W., Faghri, F., Gibbs, J.R., Singleton, A.B., Nalls, M.A., and Hernandez, D.G. (2020). Biomarkers of Parkinson’s Disease: Screening Vital Signs and Routine Blood Tests. medRxiV doi:https://doi.org/10.1101/2020.05.18.20103085

Kircher, M., Witten, D.M., Jain, P., O'Roak, B.J., Cooper, G.M., and Shendure, J. (2014). A general framework for estimating the relative pathogenicity of human genetic variants. Nat Genet *46*, 310-315.

Kocer, A., Yaman, A., Niftaliyev, E., Duruyen, H., Eryilmaz, M., and Kocer, E. (2013). Assessment of platelet indices in patients with neurodegenerative diseases: mean platelet volume was increased in patients with Parkinson's disease. Curr Gerontol Geriatr Res *2013*, 986254.

McLaren, W., Gil, L., Hunt, S.E., Riat, H.S., Ritchie, G.R., Thormann, A., Flicek, P., and Cunningham, F. (2016). The Ensembl Variant Effect Predictor. Genome Biol *17*, 122.

Pramstaller, P.P., Falk, M., Schoenhuber, R., and Poewe, W. (1999). Validation of a mail questionnaire for parkinsonism in two languages (German and Italian). J Neurol *246*, 79-86.

Qiu, X., Xiao, Y., Wu, J., Gan, L., Huang, Y., and Wang, J. (2019). C-Reactive Protein and Risk of Parkinson's Disease: A Systematic Review and Meta-Analysis. Front Neurol *10*, 384.

Sanjari Moghaddam, H., Ghazi Sherbaf, F., Mojtahed Zadeh, M., Ashraf-Ganjouei, A., and Aarabi, M.H. (2018). Association Between Peripheral Inflammation and DATSCAN Data of the Striatal Nuclei in Different Motor Subtypes of Parkinson Disease. Front Neurol *9*, 234.
